# Does a Graf Type-I Hip Justify the Discontinuation of Pavlik Harness Treatment in Patients with Developmental Dislocation of the Hip?

**DOI:** 10.3390/children9050752

**Published:** 2022-05-20

**Authors:** Yiqiang Li, Federico Canavese, Yanhan Liu, Jianping Wu, Jingchun Li, Zhe Yuan, Qinghe Zhou, Yuanzhong Liu, Weidong Chen, Hongwen Xu

**Affiliations:** 1 Department of Pediatric Orthopaedics, Guangzhou Women and Children’s Medical Center, Guangzhou Medical University, Guangzhou 510623, China; liyiq@gwcmc.org (Y.L.); doctorliuyanhan@163.com (Y.L.); gfewjp@163.com (J.W.); haiqingkun@126.com (J.L.); yuanxingzhe0502@126.com (Z.Y.); zqhetty@163.com (Q.Z.); lyzcxy@126.com (Y.L.); chweidong@163.com (W.C.); 2 Department of Pediatric Surgery and Orthopedics, Jeanne de Flandre Hospital, Lille University Center, 59800 Lille, France; canavese_federico@yahoo.fr

**Keywords:** developmental dysplasia of the hip, Pavlik harness, hip ultrasound, acetabular index, residual acetabular dysplasia

## Abstract

Background: To analyze the clinical data of patients aged < 6 months with developmental dislocation of the hip (DDH) treated with Pavlik Harness (PH) in order to identify the best time to terminate PH treatment. Method: Fifty-four patients (47 females, 7 males; 63 hips) met the inclusion criteria and were included in the study; there were 33 (61.1%) left, 12 (22.2%) right and 9 (16.7%) bilateral DDH. The mean age at diagnosis was 11.8 ± 5.9 weeks (range, 1.4–25.5). All patients underwent fulltime PH treatment for about three months. At completion of PH treatment, patients were then divided into *Group A*, including patients with clinically stable hip joint and Graf type-I hip on ultrasound (US), and *Group B*, including patients with clinically stable hip joint and well-reduced hip on anterior-posterior (AP) radiographs without acetabular dysplasia. Six months after completion of PH treatment, the presence/absence of residual acetabular dysplasia (RAD) was evaluated on AP pelvis radiographs. The *t*-test and chi-square test were used to compare the differences in age, gender, side, Graf classification and RAD rate between the two groups of patients. Results: At completion of PH treatment, 45 hips were in *Group A* and 18 in *Group B*. There were no significant differences in age, gender, side, preoperative alpha angle and Graf classification between the two groups. Six months after discontinuation of PH, the AI in *Group A* (27.1° ± 6.8°) was significantly higher than that in *Group B* (21.9° ± 3.5°; *p* = 0.001); moreover 23 hips (51.1%) in *Group A* developed RAD compared to one hip in *Group B* (5.6%; *p* = 0.001). Among *Group A* patients, those with RAD were significantly older (13.7 ± 4.9 weeks) than those with normal hips (7.6 ± 3.8 weeks; *p* < 0.001); the incidence of RAD was significantly lower in patients with Graf type-II D hips (22.2%) than in patients with Graf type-III (70%) and type-IV hips (71.4%; *p* = 0.006). However, logistic regression analysis identified age as the only risk factor for RAD. All 24 hips with RAD (24/63, 38.1%) were treated with abduction braces. At final follow-up, AI in *Group A* (20.5° ± 3.3°) was not significantly different from that in *Group B* (21.9° ± 3.3°; *p* = 0.132). At the last follow-up visit, five hips (11.1%) in *Group A* still had RAD, compared to none in *Group B* (*p* = 0.31). Conclusions: In patients with DDH treated by PH, Graf type-I on US is not an absolute timing to terminate PH treatment. In addition, patients ≥ 13 weeks had a high risk of RAD despite PH treatment as 51.1% of infants developed RAD during follow up. Follow-up radiographs should be requested in all patients achieving Graf type-I hips at completion of PH treatment.

## 1. Introduction

Developmental dislocation of the hip (DDH) is a fairly common condition in children, and successful treatment is based on early, gentle and stable hip reduction to promote the development of the acetabulum and femoral epiphysis and to avoid the onset of avascular necrosis (AVN) [1,2].

Pavlik Harness (PH) treatment is indicated in children < 6 months of age with confirmed diagnosis of DDH; the device maintains the hips at no more than 75° of abduction and 90° of flexion [2]. Generally, PH treatment is recommended for patients with Graf type IIb to Graf type IV hips, and it has very variable success rates depending on the severity of DDH, as assessed by ultrasound (US) [2,3]. In particular, the reported overall short-, mid- and long-term success rates of PH treatment range from 45% to 100% [4,5,6]. However, in patients with dislocated hips, the success rate decreases significantly, especially in Graf type IV hips [7]. Complications such as failure of reduction, femoral nerve palsy, AVN and residual acetabular dysplasia (RAD) have been reported by several authors [3,4,8].

Currently, most caregivers consider PH treatment should be discontinued when hips return to Graf type-I on US. In general, the duration of PH treatment is three months [9,10]. In clinical practice, however, we encountered some patients with Graf-I hip at the end of PH treatment and acetabular dysplasia three to six months after the end of treatment. In particular, previous studies have also reported that some patients develop RAD following successful PH treatment [11,12].

In this study, we retrospectively analyzed the clinical data of patients aged < 6 months of age with DDH treated with PH in order to identify the best time to terminate PH treatment. Specifically, we wanted to answer the following question: is a Graf-I hip an absolute indication for immediate discontinuation of PH treatment?

## 2. Materials and Methods

The medical records of children with DDH who were aged < 6 months of age at the beginning of PH treatment during the period 2014–2019 were collected and retrospectively reviewed.

The following inclusion criteria were applied: (1) diagnosis of DDH (radiographic evidence of dislocation, or US suggestive of Graf type-D, III, or IV [13]); (2) patients < 6 months of age at the beginning of PH treatment; (3) hip meeting the US or radiographic criteria for normalization upon completion of PH treatment; (4) complete clinical and radiographic data.

The exclusion criteria were: (1) failure of treatment by PH; (2) follow-up time < 12 months and/or insufficient radiographic data; (3) abnormal hip at the end of PH treatment as per US or radiographic criteria; (4) presence of underlying pathology such as cerebral palsy, spinal cord thrombosis, myelomeningocele, joint contracture and other neuromuscular diseases.

Fifty-four patients (47 females, 7 males; 63 hips) met the inclusion criteria and were included in the study; there were 33 (61.1%) left, 12 (22.2%) right and 9 (16.7%) bilateral DDH.

The mean age at diagnosis was 11.8 ± 5.9 weeks (range, 1.4–25.5). All patients underwent fulltime PH treatment for about 3 months (12 weeks); treatment started the same day of the diagnosis. The patients were evaluated once a week in the first 3 to 4 weeks to check if the hip reduced and the wearing of the PH.

### 2.1. Ultrasonography and Radiographic Assessment

Hip US was performed according to the standard method described by Graf [14]; all hip US examinations and classification of the hip were performed by pediatric radiologists with at least 5 years of experience. They all received standard training of hip US-course.

Hip US examination was performed before PH treatment, and during PH treatment at 3 to 4 week intervals, and at completion of PH treatment. The alpha angle of the hip was recorded for each hip US examination. In patients with successful hip reduction and normal hip anatomy, PH treatment was discontinued after 3 months, while in those with reduced hip and RAD, treatment was switched to an abduction brace treatment. Patients with failed PH treatment requiring closed reduction and spica cast immobilization and those with RAD at the end of PH treatment were excluded from the analysis.

Hip US or pelvis AP pelvis radiographs (supine position; non-weight bearing) were performed at the end of PH treatment in order to determine whether the hip achieved normal morphology. Forty-five hips (71.4%) underwent US examination to evaluate hip morphology; for those not able to attend US or those > 6 months of age at completion of PH treatment (n = 18; 28.6%), AP pelvis radiographs were performed.

Six months after completion of PH treatment, all patients underwent regular anterior-posterior (AP) pelvis radiographs to evaluate Acetabular Index (AI) during follow up; in addition, center edge angle of Wiberg (CEA) was measured on AP radiographs of the pelvis in patients older than 4 years of age [15]. AP pelvis radiographs were also used to evaluate the presence/absence of RAD. RAD was diagnosed if: (1) AI in patients younger than 4 years was greater than one standard deviation than the mean value for normal age matched children [16], or there was evidence of subluxation; (2) patients older than 4 years with Severin grade III or IV hips [17].

At final follow-up, AVN was assessed and graded according to the Kalamchi and MacEwen method on AP pelvis radiographs [18]. Since type I AVN is a temporary ischemic change that can be completely recovered, we considered type I AVN as normal [19].

Patients were then divided into two groups, according to the type of examination (hip US or AP radiograph of the pelvis), and hip morphology: *Group A* included patients with clinically stable hip joint and Graf type-I hip on US while *Group B* included those with clinically stable hip joint and well-reduced hip on plain radiographs with AI no more than one standard deviation above the mean value for normal age matched children [16].

Two pediatric orthopedic surgeons (LYQ and LYH) separately reviewed all AP pelvis radiographs on the Picture Archiving and Communication System of our Institution and the mean value of each measurement was used for statistical analysis.

### 2.2. Statistical Analysis

Statistical analysis was performed using SPSS 22.0 (SPSS, Chicago, IL, USA). Data included continuous numerical variables, frequencies and percentages. The *t*-test and chi-square test were used to compare the differences between the indicators of groups A and B. Logistic regression analysis (LRA) was performed to identify potential risk factors for RAD six months after completion of PH treatment. *p* < 0.05 was considered statistically significant.

## 3. Results

Of the 63 hips (54 patients), 21 (33.3%) were type-II D, 26 (41.3%) were type-III and 10 (15.9%) were type-IV according to Graf classification; the remaining 6 hips (9.5%) did not undergo pretreatment US examination, and AP pelvis radiographs revealed fully dislocated hips.

Mean age at diagnosis was 11.8 ± 5.9 weeks (range, 1.4–25.5), the mean duration of PH treatment was 3.2 ± 0.7 months (range, 2–6) and mean follow-up time was 24.4 ± 14.1 months (range, 12–61.5).

At completion of PH treatment, 45 hips (71.4%) were included in *Group A* and 18 hips (28.6%) in *Group B*.

In *Group A* patients, mean age at diagnosis was 10.7 ± 5.4 weeks, while it was 13.7 ± 7.0 weeks in *Group B* (*p* = 0.078). There were no significant differences in sex, laterality, preoperative alpha angle, Graf type or follow-up time between the two groups of patients (Table 1).

Six months after completion of PH treatment, AI of *Group A* patients (27.1° ± 6.8°) was significantly higher than that of *Group B* (21.9° ± 3.5°; *p* < 0.001). Twenty-three hips (51.1%) of *Group A* patients had RAD at six months after discontinuation of PH, which was significantly higher than that of patients in *Group B* (n = 1, 5.6%; *p* = 0.001) (Table 1). All 24 hips with RAD (24/63, 38.1%) were treated with abduction braces.

Among *Group A* patients, those with RAD were significantly older (13.7 ± 4.9 weeks) than those with normal hips (7.6 ± 3.8 weeks; *p* < 0.001); the incidence of RAD was significantly lower in patients with Graf type-II D (23.5%) than in those with type-III (70%) and type-IV hips (71.4%; *p* = 0.006) (Table 2). However, LRA found age as the only risk factor for RAD (Table 3).

SE, standard error; RR, relative risk; CI, confidence interval.

At last follow-up visit, the AI in *Group A* patients (20.5° ± 3.3°) was comparable to that of *Group B* patients (21.9° ± 3.3°; *p* = 0.132). However, at last follow-up visit five hips (11.1%) in *Group A* still had RAD, while no case of RAD was identified among *Group B* patients; although no statistically significant difference between the two groups could be identified (*p* = 0.31) (Table 1).

## 4. Discussion

Currently, there is no consensus on the duration of PH treatment in patients with DDH [7]. Weinstein et al. [15] suggested that the duration of PH treatment should be twice the patient’s age in months. Westacott et al. [7] studied the experience of 111 members of the British Society for Children’s Orthopaedic Surgery (BSCOS) in treating DDH in patients < 6 months. They found the majority of BSCOS members used PH for the treatment of DDH and the duration of treatment ranged between 6 and 16 weeks (mostly 12 weeks). Another survey by Kelley et al. [6] confirmed most pediatric orthopedic surgeons believe that PH treatment should last three months. However, this is only the personal opinion of the individual surgeon and there is no reliable evidence to show whether a PH treatment time of three months is sufficient to normalize the hip joint. The results of the present study showed that for patients with hip dislocation, an average of three months of PH treatment proved to be insufficient, as 51.1% of patients in *Group A* developed RAD even though all of them had achieved normal hip US and needed further abduction braces treatment. Dornacher et al. [12] examined 90 patients with DDH treated with PH, although US normalization of the pathological hip was achieved prior to discontinuation of PH treatment, 32.9% of hips developed RAD on radiographs taken once ambulation was acquired. Harris et al. [17] retrospectively examined 720 dislocated or subluxated hips in 550 patients treated with PH, and found that 9% of these had acetabular dysplasia at the end of PH treatment. Thus, in more severe forms of DDH, particularly Graf types III and IV, three months of PH treatment is not sufficient and longer treatment or a switch to an abduction brace is necessary to restore a normal hip morphology.

**Figure 1:**
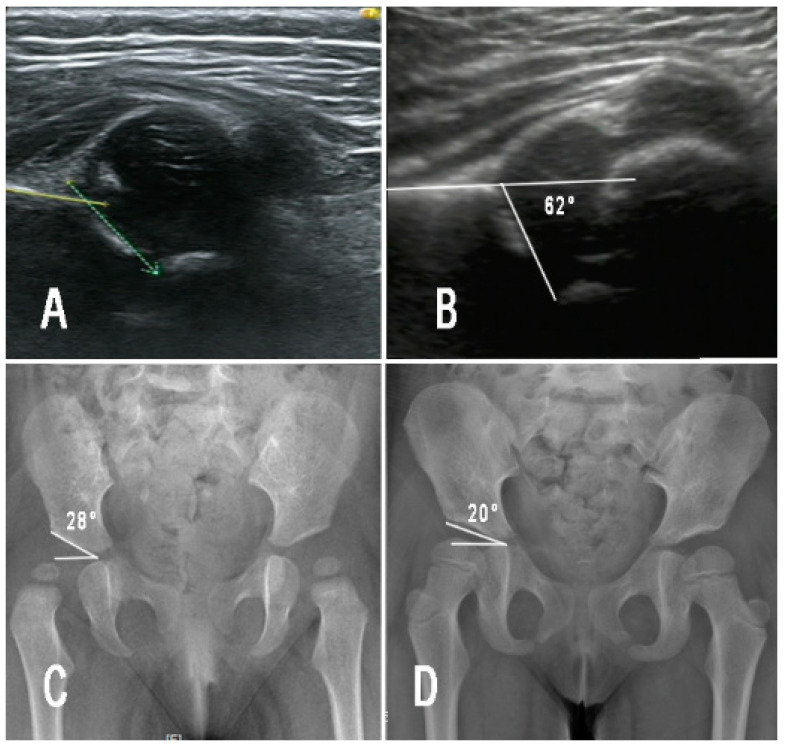
Female patient aged 3 months with right DDH (Graf type IV) (**A**), treated by PH for three months and achieved Graf type I (**B**), then the PH treatment was terminated. However, radiographic examination showed significant subluxation on the right hip 6 months later (**C**), and then the patient was treated by a night time abduction brace. At final follow-up (4 years old), the hip recovered to normal (**D**).

This study also showed that achieving a Graf-I hip is not an absolute indication to stop PH treatment (Figure 1). Currently, most pediatric orthopedic surgeons usually use US examination to determine the timing of cessation of PH treatment. In the survey by Westacott et al. [7] described above, 74% of surgeons used only US examination to assess the timing of discontinuation of PH treatment. Kelley et al. [6] surveyed pediatric orthopedic surgeons from several countries and found similar results. In our study, 45 hips with DDH achieved a Graf-I hip at the end of PH treatment, but 23 hips (51.1%) still developed acetabular dysplasia during subsequent follow-up. Similarly, Sarkissian et al. [11] retrospectively analyzed 115 patients with DDH who were treated with PH and whose hips reverted to Graf-I. Of these, 33% developed significant acetabular dysplasia on radiographs taken at one year of age. The studies by Bradley et al. [18] and Dornacher et al. [12] also showed that 16.7% to 29.4% of patients still had acetabular dysplasia at one year of age, even though US found normalization of the hip at the time of discontinuation of PH treatment.

Our data suggest that a Graf type-I hip on US does not guarantee that the hip will remain normal on plain radiographs. The reason for this may be the relatively low reproducibility of US. Despite the fact that the hip US technique developed by Graf involves rigorous examination procedures, including probe selection, patient positioning, US section selection, angle measurement and precise classification [14,19], there are still large intra- and inter-examiner discrepancies. The results of the systematic review and meta-analysis by Quader et al. [20], (n = 28 studies) suggested high variability and low agreement in all reported dysplasia metrics. Roovers et al. [21], asked five sonographers to classify 200 US images according to Graf’s method and found that the inter-observer concordance for accurate classification was k = 0.47, whereas the concordance for judging whether it was a Graf type-I was not high (k = 0.65). In contrast, radiographic examination of the hip joint has high reliability, and most studies have shown good concordance (ICC and k > 0.8) for AI and CEA measurements on AP radiographs of the pelvis [15,22]. Consequently, once treatment with PH has ended, radiographic examination should be performed to assess whether the hip joint is indeed normal. If this is not the case, it is necessary to continue treatment with PH or to switch to an abduction brace. 

This study also showed that the incidence of RAD after achieving US normalization of the hip (Graf-I) was significantly increased in patients ≥13 weeks at the time of treatment. Even though LRA failed to identify Graf type as a risk factor of RAD, among patients with Graf-III or IV hips, 70% to 71.4% still had signs of RAD 6 months after PH removal in *Group A*. Previous studies have also shown that age and Graf classification are important factors influencing the outcome of PH treatment. Novais et al. [23] studied 84 patients (134 hips) with DDH treated with PH and reported that 11.8% of these patients still had signs of RAD on AP pelvis radiographs taken at one year of age; they also noted Graf-IV hips had significantly higher incidence of RAD. Ömeroğlu et al. [24] retrospectively analyzed 130 patients (181 hips) with DDH treated with PH, and the results showed that 93% of patients < 3 months of age at the beginning of treatment evolved towards a Graf-I hip compared to only 37% of patients older than 5 months of age. Therefore, for patients ≥13 weeks at diagnosis, extra care should be taken because they are at high risk for RAD despite treatment with PH. Further studies should be performed to identify the correlation between RAD and Graf classification.

**Figure 2:**
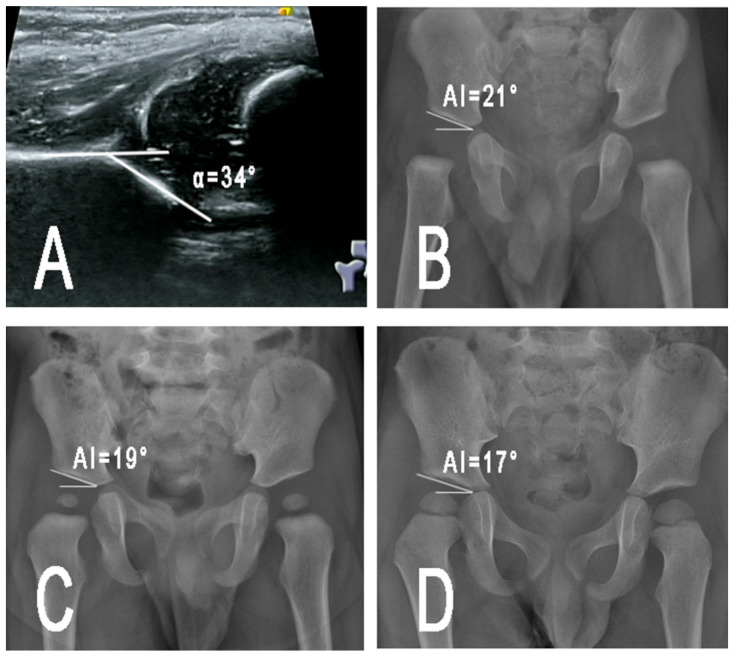
A patient aged 4 months with right DDH (Graf type III) (**A**), treated by PH for 4 months and recovered to normal radiograph (**B**). Six months later, radiographic examination showed good development of the hip (**C**). At final follow-up (2 years old), the outcome is satisfactory (**D**).

In our opinion, the variability in US results should not only be attributed to the skills of the examiners but also to the imaging examination itself: radiographs are easier to be reliably performed than US examination. Thus, patients with DDH treated by PH should undergo hip radiographic examination at the completion of PH treatment. PH should be terminated when radiographs are normal (Figure 2). In case ossific nucleus of the femoral head is not present, hip radiographs should be performed after four months of age, as suggested by the clinical guidelines of the American Academy of Orthopaedic Surgeons (AAOS) for management of pediatric developmental dysplasia of the hip in infants [25]. It is possible that when both ultrasound and radiograph are normal, PH can be safely discontinued, so as to decrease the occurrence of RAD.

## 5. Conclusions

In conclusion, in patients with DDH treated by PH, Graf type-I on US is not an absolute timing to terminate PH treatment. In addition, patients ≥13 weeks have a high risk of RAD despite PH treatment as 51.1% of infants developed RAD during follow up. If the US-results cannot be safely relied upon, follow-up radiographs should be requested at the completion of PH treatment.

## Figures and Tables

**Table 1:** Comparison of clinical data between patients achieving normal hips on hip US (*Group A*) and pelvis radiographs (*Group B*) at the end of PH treatment.

		*Group A*	*Group B*	χ^2^/t	*p*
Hips	n	45 (71.4%)	18 (28.6%)	-	-
Age (weeks)		10.7 ± 5.4	13.7 ± 7.0	1.794	0.078
Gender	Female	37 (69.8%)	16 (30.2%)	-	0.710
	Male	8 (80%)	2 (20%)		
Side	Left	25 (75.8%)	8 (24.2%)	3.359	0.239
	Right	6 (50%)	6 (50%)		
	Bilateral	14 (77.8%)	4 (22.2%)		
Preoperative α-angle		42.4° ± 4.5°	40.8° ± 6.4°	1.023	0.311
Graf classification	II-D	18 (85.7%)	3 (14.3)	1.266	0.583
	III	20 (76.9%)	6 (23.1%)		
	IV	7 (70%)	3 (30%)		
Duration of PH treatment (months)		3.0 ± 0.3	3.2 ± 0.4	1.213	0.236
Follow-up time (months)		26.0 ± 16.0	20.5 ± 6.3	1.931	0.058
RAD following removal of PH	No	22 (56.4%)	17 (43.6%)	11.314	0.001
	Yes	23 (95.8)	1 (4.2%)		
AI (6 months after completion of PH treatment)		27.1° ± 6.8°	21.9° ± 3.5°	4.045	0.000
Final AI		20.5° ± 3.3°	21.9° ± 3.3°	1.528	0.132
Final CEA		17.4° ± 6.1°	19.3° ± 4.5°	1.153	0.255
RAD at final follow-up	No	40 (69%)	18 (31%)	-	0.31
	Yes	5 (100%)	0 (0%)		

**Table 2:** Comparison of clinical data between patients with and without RAD six months after the completion of PH treatment.

		RAD		χ^2^/t	*p*
		No	Yes		
Hips	n	22 (48.9%)	23 (51.1%)	-	-
Age (weeks)		7.6 ± 3.8	13.7 ± 4.9	4.666	0.000
Gender	Female	16 (43.2%)	21 (56.8%)	-	0.135
	Male	6 (75%)	2 (25%)		
Side	Left	11 (44%)	14 (56%)	-	0.239
	Right	5 (83.3%)	1 (16.7%)		
	Bilateral	6 (42.9%)	8 (57.1%)		
Preoperative α-angle		43.6° ± 4.7°	41.1° ± 4.0°	1.901	0.064
Graf classification	II-D	14 (77.8%)	4 (22.2%)	-	0.006
	III	6 (30%)	14 (70%)		
	IV	2 (28.6%)	5 (71.4%)		
Duration of PH treatment (months)		3.1 ± 0.3	3.1 ± 0.4	0.075	0.94

**Table 3:** Logistic regression analysis to evaluate risk factors for RAD.

	Coefficient	SE	Wald	*p*	RR	95% of CI for RR
Age	0.179	0.064	7.927	0.005	1.196	1.056, 1.355
Graf type	0.564	0.447	1.593	0.207	1.758	0.732, 4.222

## Data Availability

Data are available from the corresponding author.
